# Do syngameons exist in tropical trees? Challenges to determine their existence and estimate their frequency

**DOI:** 10.1098/rsbl.2025.0444

**Published:** 2025-11-05

**Authors:** Sandra Cervantes, Rowan Schley, Olivier J. Hardy, Dario Ojeda

**Affiliations:** ^1^ Department of Forest Sciences, University of Helsinki, Helsinki, Finland; ^2^ Finnish Museum of Natural History, University of Helsinki, Helsinki, Finland; ^3^ Department of Geography, University of Exeter, Exeter, UK; ^4^ Department of Evolutionary Biology and Ecology, Université Libre de Bruxelles, Brussels, Belgium

**Keywords:** reticulate evolution, syngameon, phylogenomics, hybridization, introgression, tropical forests, gene flow

## Abstract

Syngameons consist of a group of species interconnected by repeated cycles of gene flow, where interbreeding can lead to fertile hybrid offspring capable of backcrossing with the parental species, facilitating the introgression of genomic regions among species. These networks have been known for over a century in plants, although mainly documented in temperate tree species. Emerging evidence from phylogenomic studies strongly suggests the existence of syngameons in tropical rainforest trees, challenging the traditional view that tree species hybridization is rare in these tropical ecosystems. Several biological characteristics of tropical trees, such as high number of co-occurring species in tropical ecosystems, generalist pollination strategies and predominantly outcrossing, could favour the evolution of syngameons. Here, we review the most recent approaches to distinguish the signal of hybridization from other evolutionary processes. We emphasize the need for robust methodologies to detect hybridization and introgression, advocating for an integrative framework combining phylogenomic, phylogeographic and population genomics analyses that will allow us to confirm the presence of syngameons in tropical trees. We argue that syngameons in tropical ecosystems may play a significant role in the distribution and maintenance of species diversity and discuss that a more integral approach of conservation is required to safeguard the integrity of these networks

## 1. Introduction

Reproductive isolation was, until recently, seen as a defining aspect of species [1]. However, a growing body of recent genomic evidence suggests that hybridization (interbreeding between different species) and resulting gene flow are important evolutionary forces. Syngameons are multispecies networks connected via gene flow [2] in which hybridization between any two different species can produce fertile hybrid offspring. In many cases, the hybrid offspring can reproduce with one of the parental species (backcrossing), resulting in the transfer of genetic material across species boundaries (introgression). Under this scenario, most fitness costs inherent within hybrid offspring do not have lasting evolutionary effects if the hybrid can survive and reproduce, thus acting as a conduit for the flow of genetic material between the parental species. The syngameon concept was first introduced in 1925 by Gunnarson [3] to explain repeated cycles of hybridization and backcrossing among a group of birch tree species. Although the species involved in syngameons are connected by gene flow, they still retain their species phenotypical distinctiveness. Schmitt *et al*. [4,5] suggest that syngameons seem to evolve both at the species levels by optimizing adaptive fitness to the ecological niche and at the syngameon level benefiting all the species involved through adaptive introgression.

Since the first introduction of the concept [3,6,7], syngameons have been reported in several plant families [8]. In tree species, syngameons have been frequently documented and studied in species of temperate regions, e.g. *Populus* [9–13], *Picea* [14,15], *Salix* [11]*, Pinus* [16,17], *Arbutus* [18], *Eucalyptus* [19,20] and *Quercus* [21–24]. Emerging evidence from these studies suggests that participation in a syngameon could provide alternative mating opportunities [25]. Additionally, access to these networks may be selected for due to positive fitness effects, such as escape from Allee effects [26] or adaptive introgression [27]. This could be particularly relevant for species with low population densities [25], in which hybridization among members of a syngameon may reduce extinction risk by diminishing the probability of inbreeding and the accumulation of deleterious mutations [8,21,25,28–30]. However, there remains unknown relevant aspects about syngameons such as how these hybridizing networks are maintained over time, how selection and recombination shape the genomic landscape of introgressed regions, what proportion of the genome is shared among the species involved and the proportion of the genome that maintains species identity or how frequent introgression contributes to adaptation.

The tree floras of tropical rainforests are the most species-rich on Earth [31], with some communities hosting more than 650 species in a single hectare [32], which results in high levels of coexistence among related tree species. Such an extraordinary level of sympatry, coupled with the outcrossing mating systems, generalist pollination and overlapping phenology of many tropical tree lineages, should mean ample opportunity for the formation in syngameons. However, there remains a lack of conclusive evidence demonstrating the presence and frequency of syngameons in tropical rainforest tree communities. Traditionally, hybridization and gene flow among closely related tropical rainforest tree species was considered rare or non-existent [33,34] because hybridization among trees in tropical ecosystems was seen as disadvantageous. This line of reasoning was influenced by the fact that species from tropical rainforests display high levels of niche partitioning and specialization, which led to the assertion that hybridization must therefore result in maladaptation—in other words, that hybrids are poor competitors [33,35]. However, some of the conclusions reached by these authors were not drawn from the empirical testing of reproductive isolation among species but instead based on their personal understanding of species ecological relationships in tropical ecosystems. Moreover, from a classical taxonomic point of view, hybridization in tropical trees is considered rare due to the paucity of observed intermediate morphologies [33,35,36].

Hybridization has been traditionally considered detrimental, as it was thought that it could hamper the process of speciation [37]. The prevailing view was that for the process of speciation to be completed, incipient species should reach reproductive isolation [1]. Lately, there has been a shift from considering hybridization as detrimental and infrequent towards recognizing its frequency and its relevance in driving plant evolution [38,39]. This shift in perspective has been facilitated by the advent of genomic data, which allows the sampling of larger portions of the genome revealing the prevalence of hybridization events and introgressed regions, thus supporting the genic view of speciation [40]. A similar trend has been observed in tropical tree species [5,41–51], something already suspected based on observed patterns of geographically structured chloroplast capture [52–57], which suggests that tropical trees might form syngameons.

It is important to note that not all the instances of hybridization represent syngameons. The key aspect to identify syngameons is to distinguish the signal of ‘occasional’ hybridization from that of introgression events that might indicate repeated cycles of hybridization-backcrossing-introgression. These cycles are likely variable in space and across time periods and depend on fluctuating ecological conditions that can favour genetic exchange [25]. This is relevant because we know that: (i) syngameons are rare relative to the total number of hybridization events detected in plant lineages [8] and (ii) our understanding of the relevance of introgression in tropical trees is still incomplete as sampling strategies in phylogenetic analyses correctly represent the species, but not its distribution range. Therefore, the potential extent of hybridization events across the landscape it is not fully captured. As the availability of phylogenomic datasets and the representation of the distribution range of species improves [41,58], we will be in a better position to determine the extent and frequency of tropical tree syngameons. If true, their existence could have important implications in our understanding of the processes that maintain species diversity in tropical ecosystems.

Here, we provide an overview of current knowledge regarding the preconditions that can favour the existence of syngameons in tropical trees by discussing the biological characteristics that might facilitate hybridization, introgression and the maintenance of syngameons. We then review and discuss current approaches and methods to differentiate hybridization and introgression from other evolutionary processes. Finally, we propose an integrative framework to confirm the existence and extent of tropical syngameons and discuss the possible implications of these entities for diversity estimation and species conservation.

## 2. Factors that could favour hybridization and the formation of syngameons in tropical trees

Tropical regions harbour the highest number of co-occurring tree species on Earth. Current estimates indicate the existence of *ca* 37 900 described tropical tree species worldwide [31,59]. The most relevant feature is the large numbers of closely related species coexisting in sympatry, which in principle can create the conditions conducive to hybridization events. Estimates from tropical forest inventories have reported more than 800 tree species (≥10 cm dbh) in 25 ha within Lambir Hills in Borneo and in the Yasuní National Park in Ecuador. Even smaller plot sizes contain many tree species in sympatry, for example up to 655 tree species have been recorded in 1 ha of tropical rainforest (≥10 cm dbh) in Amazonia [32,60]. Often, many closely related species of the same genus coexist in sympatry. For instance, 19 *Inga* species (Leguminosae) can coexist in 1 ha of tropical rainforest in western Amazonia [61], 11 species of *Macaranga* (Euphorbiaceae) have been reported in sympatry in Borneo [62] and up to 15 species of *Eschweilera* (Lecythidaceae) have been found to coexist in Central Amazonia [63].

Another feature of tropical trees is their relative abundances over the landscape, i.e. some species are extremely rare, and some others are hyperabundant. The spatial distribution and abundances of tropical trees seem consistent with the presence of ‘hub species’ observed in temperate tree syngameons, which connect less abundant species across the landscape via asymmetric gene flow [29]. In tropical forests, syngameons could be selectively advantageous for very rare species as participation in a syngameon could allow mating events with the other species in the syngameon, increasing their effective population size and reducing their probability of extinction [30]. In Amazonia, for instance, only a small proportion of tree species are considered common and abundant. These species constitute at least 50% of all individual trees in a specific sampled area and represent a ubiquitous feature of tree communities at local and regional scales. These abundant tree species are referred to as oligarchs in Western Amazonia [64] or hyperdominant in lowland Amazonia [65]. These oligarchs and hyperdominant species represent only 1.4% of the estimated 16 000 tree species in the Amazonian rainforest [65], while at the global level about 2.8% of tropical trees are considered hyperdominant [31]. This feature also points towards the opposite side of this pattern, indicating that *ca* 93% of the tropical tree species are considered less common or rare species. This has led to the pattern of ‘rare is common and common is rare’ in tropical trees [65]. The eco-evolutionary processes that maintain hyperdominance and the existence of species with low population densities (rare species) is still not fully understood, and it is currently an active area of research [66].

In addition to the high levels of sympatry, a high proportion of tropical trees are predominantly outcrossing [25,67], rely on generalist pollinators [68–70] and have overlapping phenology [71], which could promote hybridization and introgression among closely related species. The suggestion that tropical trees with low abundance might have mating behaviour flexibility, relying primarily on an outcrossing strategy [71] but maintaining a diminished capacity for hybridization with closely related species, could also favour the formation and maintenance of syngameons [25]. Also, some studies have indicated that similar ploidy levels among closely related species give them an evolutionary advantage by allowing them to be involved in syngameons [58,72–75]. However, there is emerging evidence that hybridization can occur between species with different ploidy levels and that polyploids can function as gene flow bridges among diploid species that cannot hybridize [76–79], something we consider could aid the formation of syngameons.

## 3. Approaches to detect hybridization and the challenges to identify putative syngameons

**Figure 1.**
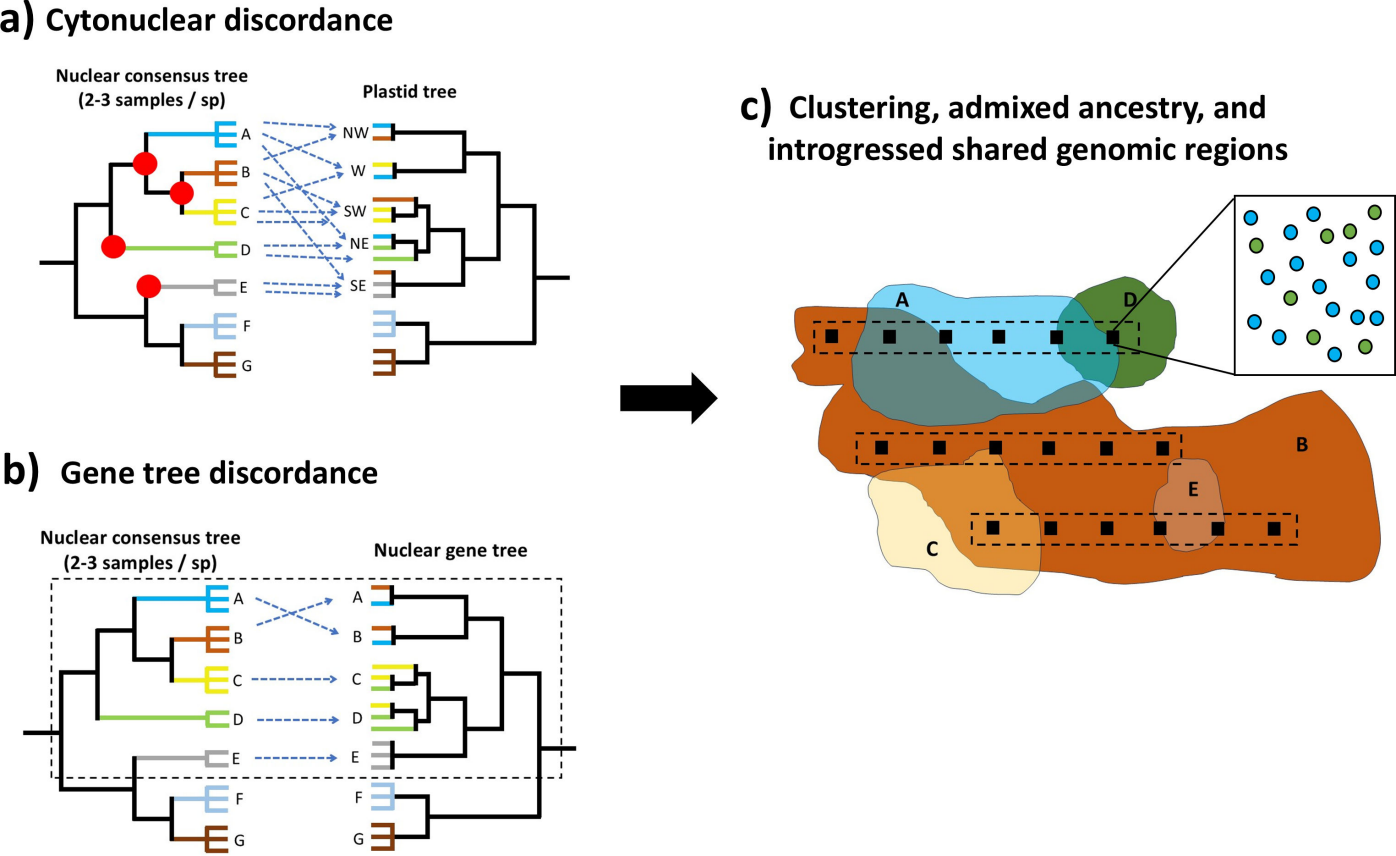
Proposed strategy to identify hybridization events between species (A to G) and the extent of putative syngameons using a combination of phylogenomic, phylogeographic and population genomic approaches. (a) First using cytonuclear discordance, where the red dots indicate the location of hybridization events in the phylogenetic tree and the geographic information of the specimens (symbolized by cardinal directions: NW, W, SW, NE, SE) suggest the extent of the putative syngameon (here, species A to E). (b) Analysing gene tree discordance to identify the species involved in the putative syngameon and the tentative direction of gene flow. (c) A localized sampling of transects along the distribution of the potential species involved in the syngameon with multiple specimens/species to identify the direction of introgression and the candidate genomic regions shared. Each colour and letter represents a distinct species in the phylogenetic trees (represented by multiple individuals). The black squares represent plots of a specific size (e.g. one hectare) that will depend on the distribution of each species. The different dots inside the square represent different tree individuals of each species, with the different colours representing different species.

The main signal that indicates the existence of putative syngameons in plants is the presence of recurrent hybridization events, often between non-sister species, in a phylogenetic analysis [54]. This is usually inferred with the discordance in tree topologies obtained from different genomic markers: between the plastome versus nuclear (cytonuclear discordance) and with the discordance among nuclear genes (gene tree discordance). Current strategies to detect hybridization first involve the identification of cytonuclear discordance by comparing the reconstructed topologies from the plastome and a set of nuclear genes [80,81]. The topologies obtained from the two datasets are analysed visually, for example using a tanglegram [82]. In syngameons, the plastome topology tends to give a phylogeographical signal (i.e. lineages tend to segregate by geographic region (figure 1a), providing evidence of the extent of possible hybridization events, while the nuclear genes usually reflect taxonomic boundaries (delimitation of species). However, we still have a limited representation of the extent of cytonuclear discordance across plant systems as the combined analyses of plastome and nuclear genes is still becoming the norm in genomic datasets (table 1).

**Table 1.** List of genera of tropical tree species where hybridization events and the existence of putative syngameons have been reported or suggested. Instances of deep hybridization events is indicated in bold. *The total number of species was based on data from Plants of the World Online (https://powo.science.kew.org/) and from our own estimates in the Leguminosae.

family	**genus** species sampled/total*	**geographic region**	**cytonuclear discordance**	**nuclear gene tree discordance**	**syngameons**	**reference**
Lauraceae	*Caryodaphnopsis,* 10/16	China, Vietnam	yes	—	—	[80]
Lecythidaceae	*Eschweilera,* 38/94 *Lecythis*, 5/5	central and south tropical America	yes	—	yes	[5,83]
Rubiaceae	several genera	cosmopolitan	yes	—	—	[53]
Melanthiaceae	*Paris,* 29/29	Eurasia	yes	—	—	[57]
Annonaceae	*Oxandra,* 20/29	tropical America	yes	—	—	[84]
Annonaceae	*Dasymaschalon,* 21/29 *Friesodielsia,* 13/50	subtropical Asia	yes	—	—	[43]
Proteaceae	*Adenanthos,* 30/34	Australia	yes	—	—	[55]
Myrtaceae	*Syzygium,* 182/1246	tropical and Subtropical Old World to Pacific	yes	yes	—	[58]
Sapindaceae	*Xanthoceras,* 1/1	northern China, Korea	yes	yes	—	[85]
Malvaceae	*Adansonia,* 8/8	tropical and southern Africa, Madagascar, southern Arabian Peninsula, NW. Australia	yes	yes	—	[86]
Leguminosae	*Brownea,* 23/27	Neotropical	—	yes	yes	[43]
Leguminosae	*Inga,* 189/294	Neotropical	—	yes	yes	[36]
Asteraceae	*Commidendrum*, 4/4 *Melanodendron*, 1/1	St Helena	yes	—	yes	[87]
Arecaceae	*Ceroxylon*, 6/13	southern America	—	yes	yes	[88]
Rutaceae	*Zanthoxylum*, 44/235	cosmopolitan	—	yes	—	[89]
Clusiaceae	*Symphonia*, 3/16	Mexico to tropical America, tropical Africa, Madagascar	—	—	yes	[4,5,50]
Fagaceae	*Castanopsis*, 12/145	tropical and subtropical Asia	—	yes	—	[90]
Ebenaceae	*Diospyros*, 12/787	cosmopolitan	—	yes	—	[47]
Leguminosae	*Brachystegia*, 28/32	tropical and southern Africa	yes	—	yes	[91–93]
Leguminosae	*Eperua*, 19/19	tropical America	yes	yes	—	[94]
Moraceae	*Ficus*, 232/881	tropics and subtropics	yes	yes	yes	[95,96]
Myrtaceae	*Eucalyptus*, 22/716	Philippines to Australia	yes	yes	yes	[20]
Leguminosae	*Sindora*, 20/20	central tropical Africa, tropical and subtropical Asia	yes	yes	yes	[97,98, unpub.]
Leguminosae	*Anthonotha*, 17/17 *Englerodendron*,17/20 *Berlinia*, 21/21 *Isoberlinia*, 4/5	tropical Africa	yes	yes	yes	[unpub.]

**Figure 2.**
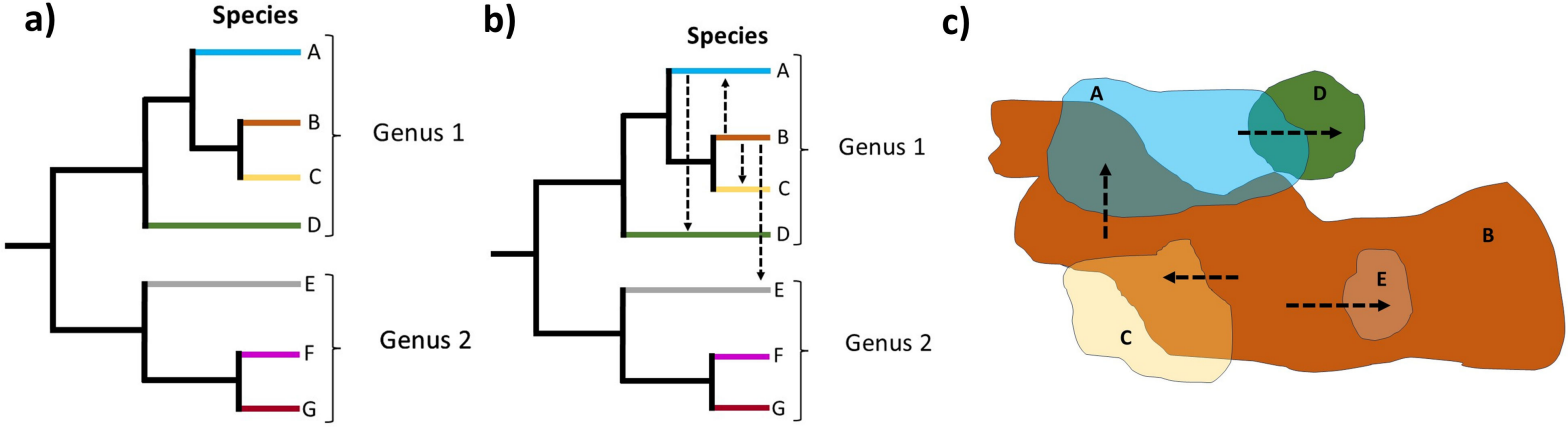
(a) Bifurcating pattern of phylogenetic relationships of seven species (different colours in A–G) from two genera without representing the existence of hybridization events. (b) Network representation showing the hybridization events, direction of gene flow and the taxa involved in the syngameon. (c) Schematic representation of the distribution range of the syngameon. The arrows indicate the direction of gene flow within the syngameon. Each colour and letter represent a distinct species in the phylogenetic trees (represented by multiple individuals). Species B is considered a hub species, connecting other species in the syngameon.

Another limitation is that most phylogenomic analyses only include a limited number of specimens per species. This representation usually does not capture the full geographic distribution of each species, which limits our capacity to assess the broader geographical distribution of hybridization events at the landscape [99–101] (figure 2). However, it is important to note that not all the signal of discordance is indicative of hybridization events, as other processes, such as incomplete lineage sorting (ILS or the retention of ancestral polymorphisms during speciation), paralogy due to genome duplications, horizontal gene transfer [102,103] and methodological errors can generate similar signals [52,103]. Here we consider that the main challenge is to distinguish discordance signals originating from ILS from those of true hybridization events.

Once cytonuclear discordance is detected and hybridization is suspected, gene tree discordance can be used to further distinguish between hybridization and ILS. This approach involves comparing the species tree against the multiple topologies obtained from individual nuclear genes (figure 1b). Under ILS, we expect an even ratio of all discordant topologies, given that ILS acts randomly across all members of a clade that originated from a common ancestor. In contrast, gene flow in a limited number of species will generate a bias towards a particular discordant topology. Several programmes have been developed to visualize and/or quantify gene tree discordance, such as PhyParts [104], DiscoVista [105] and Phytop [106].

Two main categories of methods have been developed to distinguish ILS from hybridization [107–109]: (i) summary methods based on a bifurcating tree topology and (ii) phylogenetic networks that represent reticulate evolution of unrooted (splits) or rooted (reticulate) networks (Table 2). Summary methods use gene trees or aligned sequences as an input and can identify hybridization events in subsets of three [119,120], four [140] or five taxa [108,116,117]. Summary methods are advantageous in their speed and scope, as it is possible to implement them in large phylogenomic datasets. By combining different summary methods, it is possible to identify hybridization events, their direction [80] and the timing of their occurrence [82]. The main disadvantage of summary methods is their inability to reconstruct and represent reticulate evolution (or non-bifurcating relationships) (figure 2a). It is becoming more evident that certain phylogenetic relationships are not captured by a bifurcating tree, and it is better to represent them via networks [141] (figure 2b–c).

**Table 2.** List of some of the programmes used to infer hybridization within a phylogenomic framework.

**category**	**input**	**method/platform**	**inference**	**reference**
summary methods	gene trees	MSCquartets	coalescence	[110]
		TICR	coalescence	[111]
		Aphid	approximate maximum likelihood	[97]
		Quartet Sampling	maximum likelihood	[112]
		QuIBL	coalescence	[113]
		DiscoVista	coalescence	[105]
		JML	coalescence	[114]
	aligned sequences	HyDE	phylogenetic invariants	[115]
		D_FOIL_	phylogenetic invariants	[116]
		Δ-statistics	coalescence	[117]
		Patterson D	coalescence	[118]
		D3	coalescence	[119]
		theta	coalescence	[120]
		ALPHA	phylogenetic invariants	[121]
		Dp	coalescence	[122]
phylogenetic networks	unrooted trees	PhyloNetworks	maximum pseudolikelihood	[123,124]
	biallelic rooted trees aligned sequences	PhyloNet	maximum pseudolikelihood maximum parsimony Bayesian maximum pseudolikelihood	[125]
	gene trees	NANUQ	coalescence	[126]
		STEM-hy	maximum likelihood	[127]
	rooted trees	PIRN	maximum parsimony	[128]
		Dendroscope	maximum parsimony	[129]
	aligned sequences	NetRax	maximum likelihood	[130]
		BPP	Bayesian	[131]
		SpeciesNetwork	Bayesian	[132]
		PhyNest	composite likelihood	[133]
		BEAST2	Bayesian	[134]
		SnappNet	Bayesian	[135]
	allele frequency data	MixMapper	pseudolikelihood	[136]
		TreeMix	pseudolikelihood	[137]
	aligned sequences	Splitstree	agglomeration	[138,139]

Phylogenetic networks overcome the bifurcating assumption and can infer complex reticulation events [109,142]. However, these methods are computationally demanding and are only applied to small datasets, thus limiting their use to a narrow set of taxa. For instance, phylogenetic network methods that use the full likelihood under the multispecies network coalescence can only be applied to fewer than 10 taxa and three reticulation events [109]. Also, some of these phylogenetic network methods require a prior specification of the number of hybridization events, which can restrict their application. One exception to this limitation is some methods that use a heuristic approach to provide an estimate of hybridization events based on the pseudolikelihood, for example with PhyloNetworks [123] or with the use of tools such as OptM that can infer the optimal number of migrations events (hybridization edges) in Treemix [143]. There are currently a limited number of benchmarking studies comparing these two main categories of methods [108,116,120,131,144,145]. Furthermore, both summary and phylogenetic network methods seem to be over-sensitive to multiple and deeper hybridization events, causing an overestimation of the amount of reticulation in a clade [108]. Conversely, tools such as PhyloNetworks [123] are prone to underestimate the number of hybridization events because they can fit only one hybridization event on a branch and so multiple hybridization events involving the same lineage cannot be inferred [124].

Another challenge is to distinguish if the discordance signal comes from an ancient or a recent hybridization event. Ancient hybridization is an event that occurred in the past and it has concluded, resulting in a novel and stable lineage [38]. In contrast, recent hybridization involves ongoing gene flow among species. Recent hybridization can generate a phylogeographic signal independent of species boundaries at introgressed nuclear regions, in contrast to ILS, because the alleles shared between introgressing species may not have had the time to spread across the distribution ranges of each species (figure 1b). Recent hybridization can be shallow, hybridization between species in the same genus, or it can occur between species from two deeply divergent lineages, i.e. between genera (intergeneric, deep hybridization) [43]. To date, most of the known hybridization events in tree species appear to be shallow, while deep hybridization events are considered rare [38]. Deep hybridization has been reported in a few instances in temperate trees [146,147] and is only known in a few instances in tropical trees in Leguminosae, Annonaceae and Asteraceae (table 1). The most recent phylogenomic analyses reporting ancient and deep hybridization events have been based mainly on exome capture [99,100,148], but analysis based on whole genomes are becoming more common [58,75,149–152].

## 4. An integrative framework to confirm the extent of syngameons

**Figure 3.**
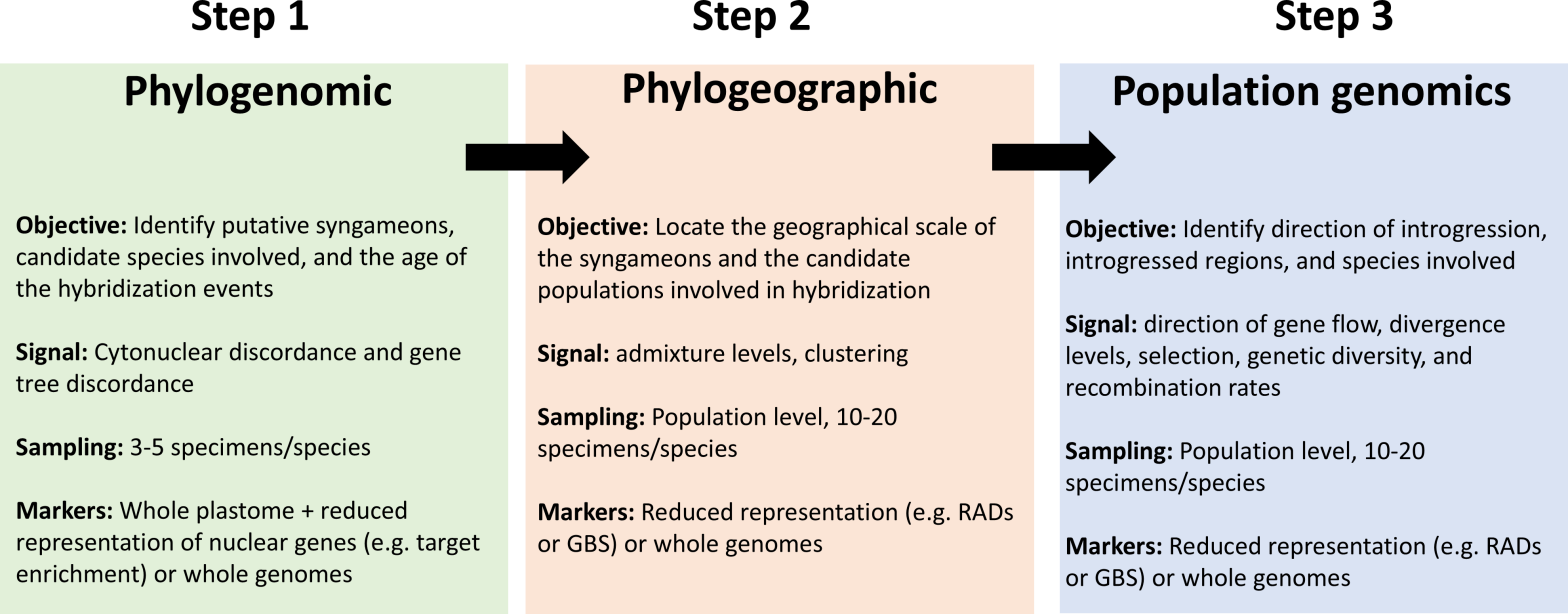
Flowchart indicating the main steps and components of the proposed integrative framework divided into a phylogenomic, phylogeographic and population genomics.

We consider that an integrative framework to determine the phylogenetic and geographic extent of syngameons should involve a combination of phylogenomic, phylogeographic and population genomics analyses at multiple scales in the landscape (figure 3). First, we suggest a phylogenomic approach using a combination of summary and phylogenetic network methods together [142] with a strategic sampling to establish their phylogenetic extent [44]. Sampling should include multiple individuals for each species, representing as much as possible their distribution range. This is important for widely distributed species, which might be hybridizing with different species at different locations of their distribution. This will also increase the likelihood of capturing hybridization events occurring at the different branches of the phylogeny. This first step will provide the phylogenetic position of hybridization and introgression events (putative syngameons) in the group of interest and the candidate species involved. At this level, it is possible to discern between ancient and deep hybridization events. One suitable approach is to estimate the average of F_4_ ratios along the branches in the phylogeny with fbranch [118] and locate the introgression events on internal branches, providing a temporal scale of the events. Such an approach has been employed to identify the conflicting signal generated from ancient introgression events in *Populus* and *Salix* [11]. One recommendation of the inference of introgression events at this level is to use closely related taxa as outgroups in the analyses and to explore the possible effect of ghost lineages (unknown, unsampled or extinct) that might lead to incorrectly identifying the donor and recipient species [153].

Second, a combination of a phylogeographic and population genomics approaches with a more intense sampling at a finer geographical scale is required to confirm putative syngameons from the previous step [6,154–156]. The sampling requires the availability of geographical areas where the location and identity of co-occurring tree species have been established and where multiple individuals can be collected, e.g. within established forest plots [157]. Multiple transects can be established across the landscape, capturing the distribution and overlap of the candidate species involved in the syngameon (figure 2c). Ideally, the combination of these transects across the landscape will provide enough scope to capture the geographical areas where the species could be introgressing, thus enabling the identification of the geographic extent of the syngameon. To our knowledge, such a sampling approach has not been applied in any tropical tree group [4,49,50,158]. Some studies have used a similar strategy in localized geographic areas in temperate and subtropical trees [17,21,24,154]. However, we recognize that this sampling strategy can be challenging in remote and underexplored tropical regions. Researchers can make use of databases [159] as a head-start to establish species distribution ranges. Access to herbarium specimens can also be used to aid the establishment of distribution ranges and to complement the sampling from currently inaccessible regions. Deeper understanding of the evolutionary mechanisms influencing syngameons at the finer scale requires a population genomics approach with markers ideally obtained from whole genome sequences. However, other cost-effective strategies, such as reduced representation of the genome (RADs or GBS) can also be used, albeit with some limitations regarding the amount of the genome that can be sampled, which can decrease the likelihood of identifying introgressed regions.

We suggest that at least three lines of evidence from population genomics can provide support for the existence of tropical tree syngameons: (i) the identification of individuals with multiple levels of mixed ancestry from two or more parental species, reflecting that introgression is occurring frequently, rather than representing occasional hybridization events. Identifying these individuals with an admixed ancestry across the landscape could be challenging, considering that introgression events might occur at low frequency and at intermittent periods of time [160]. The identification of the species involved in a syngameon require admixture and genetic clustering analyses to identify shared ancestry (e.g. D statistics, F_4_ ratios), and the identification of the direction of the introgression events requires multi-tip extensions of the D statistic, such as D_FOIL_ [116,117,161]. To discern this pattern from a hybrid zone between only two species, it is necessary to identify multiple species with evidence of introgression, perhaps forming several hybrid zones across the landscape. To date, such an approach has not been carried out in tropical trees and only a couple of studies have analysed putative syngameons at the population level, focused on a pair of species [49], or on a set of closely related species [88].

(ii) the identification of shared introgressed genomic regions among the species involved in the syngameon, especially when they are geographically restricted to specific contact zones. We suspect that the direction of introgression events and gene flow should be influenced by the abundance, geographic location and distribution of the involved species in the landscape (figure 2c). Given the unequal abundances of tropical tree species, we expect to observe asymmetric gene flow (introgression) from the most abundant species into those species with lower abundance. Also, the most abundant and widespread species should have more introgression opportunities with different species across their distribution range. One of the main challenges of identifying these shared genomic regions is the scarcity of genomic resources in tropical trees such as reference genomes and linkage maps. To increase the likelihood of identifying introgressed regions, it is preferable to obtain whole genome sequences from multiple individuals for all the species involved [27,162].

Identifying shared genomic regions and barriers to gene flow remains challenging given the variety of evolutionary forces (migration, selection and linkage) that generate genetic diversity and divergence along the genome. We propose the application of two complementary approaches currently used in speciation genomics to identify these barriers and the shared genomic regions within the syngameon by (i) exploiting the genome-wide relationship between the location of introgressed regions and recombination rates, (ii) by applying demographically explicit genome scans for barriers to gene flow [163], and iii) by identifying areas in the genome with reduced nucleotide diversity. (i) Location of introgressed regions and levels of recombination. Two main expectations have been suggested regarding the associations between the location of introgressed regions and recombination rates, either located in areas with high recombination or in areas with low recombination rates [154]. If the introgressed regions carry mainly deleterious effects they are expected to be situated in areas of the genome with high recombination rates. Therefore, adaptive or neutral variation linked to the introgressed region can be recombined and disassociated from these mainly deleterious regions. This has been the conventional view, as gene flow and introgression have been considered mostly with deleterious effects. Such a pattern has been reported for introgressed regions in *Populus* [13,164] and *Castanopsis* [90] species, where introgressed regions were in areas of the genome of high recombination rates. However, more recent theoretical work suggests that the time of divergence between hybridization species has an influence on these associations. The fitness effects of large introgressed regions might be neutral or mainly beneficial, rather than deleterious in recently diverged species, thus facilitating the introgression and the maintenance of large genomic regions [165]. These introgressed genomic regions are more likely to be maintained by selection due to their fitness effects and are expected to be in genomic regions with low recombination rates. This expected correlation has been reported in *Quercus* [23] and *Populus* species [27], where introgressed regions were preferentially situated on regions of low recombination rates. As more genomic datasets become available for tropical trees, it will eventually be possible to apply the most recent machine learning approaches that incorporate population genomic data to identify introgressed regions and further dissect their adaptive role [166–169]. (ii) Performing demographically explicit scans for barriers to gene flow (e.g. gIMble) [163] by assessing the fit of isolation-with-migration and strict-isolation demographic models. Genomic windows in which strict isolation is the best-fitting model are those that act as barriers to gene flow [162]. While under this statistical framework, it is possible to identify the position and the strength of barriers to gene flow, this approach is most suitable for recently divergent taxa. iii) Recent adaptive introgressed regions tend to have reduced nucleotide diversity compared to the genomic background [27]; hence, this characteristic pattern can be used for their identification using e.g. VolcanoFinder [170]. This tool identifies regions in the recipient species with low heterozygosity (due to selective sweeps on introgressed variation), flanked by two ‘peaks’ of heterozygosity that result from the introgression event [170]. This approach has the advantage of identifying adaptive introgressed regions in the recipient species without needing to sample the donor species.

The previous approaches will identify evidence of selection acting of the introgressed regions but do not unequivocally provide evidence of the adaptive role of the introgressed regions. Thus, these regions are more correctly referred to as putatively adaptive, demonstrating the adaptive role of these requires linking them to phenotypic variation and fitness, something that has been reported in very few instances in plant species [28].

## 5. The implications for species diversity estimation and the consequences for the conservation of tropical trees

Current estimates indicate that there are 37 900 described tree species in the tropical region [37], with extrapolations suggesting the existence of *ca* 47 000 tropical tree species [31]. Species biodiversity estimates, extinction risk evaluation and prioritization for conservation are done at the species level. Hybridization is mainly perceived as a threat under the current view of conservation biologists and policy makers, due to the potential effects of outbreeding depression and extinction of lineages [37,171]. This perception has been influenced by the idea that each tree species evolves independently and the depiction of phylogenetic relationships as bifurcating patterns (figure 2a). Under this understanding, the unit of conservation is at the species level, but if syngameons are more common than we suspect in tropical trees, we will need to rethink what we consider as the main evolutionary unit and to re-assess conservation priorities at the species level. For instance, abundant and common (i.e. hyperdominant) species are considered less threatened than rare species. However, if tropical trees are structured as syngameons, hyperdominant and widespread (hub) species connecting the network across the landscape (figure 2c) might have far more important roles for the long-term survival of less common species [21,25,30]. For example, *Gilbertiodendron dewevrei* (Leguminosae) is widely distributed in the Congo basin across Nigeria, Cameroon, Central African Republic, Gabon, the Republic of Congo and the Democratic Republic of Congo (DRC). This species is abundant and forms monodominant stand forests interspersed within mixed terra firme forest across the landscape [172,173]. Given its distribution and abundance, this species is not considered endangered, but it is logged, and it has commercial value in the Congo basin. We argue that species with these characteristics, such as *G. dewevrei*, could have important roles in sustaining biodiversity, not only for its ecological role structuring the community, but for its potential role connecting less abundant species via introgression as a ‘hub’ species in syngameons. Rare species could also have prominent roles, as these species could be more likely to generate heterospecific crosses and transgressive (extreme) phenotypes within syngameons [25] (figure 2c), potentially affecting diversification rates.

Shifting our understanding of the role of abundant and rare species can increase our knowledge of the mechanisms explaining the distribution of diversity in species-rich ecosystems, i.e. many species with low abundance and lower than expected diversity of the proportion of genera [29]. A conservation perspective that highlights the syngameon as the evolutionarily relevant unit has been suggested for North American oaks [25,38,174], European oaks [175], *Betula* species [6] and a putative syngameon in the *Commidendrum—Melanodendron* (Asteraceae) of St Helena [87]. For species where the extent of the syngameon is unknown an approach based on distribution data can be a first approximation of their potential connectivity, like it has been obtained for the North American oaks [38]. This information could be combined with the individual species abundances and their IUCN status for a more comprehensive conservation assessment. Furthermore, we need to reconsider the evolutionary role of hybrids in these species-rich ecosystems. It has been suggested that hybrids can function as potential genomic bridges among partially interfertile species and as a possible reservoir of advantageous alleles [160,176]. Thus, their evolutionary implications for biodiversity conservation might have long-lasting impacts of which we are not yet completely aware.

## 6. Concluding remarks and recommendations for future research

Despite the increasing number of phylogenomic analyses reporting hybridization events in several tropical tree species, we still lack conclusive evidence of the evolution of syngameons in these lineages. Hybridization events are the first step to further explore how common syngameons are, but our current sampling strategy and analyses are limited. Our current focus is on species pairs of hybridizing species in the syngameon, and we require more strategic sampling to capture the complex patterns of interspecific hybridization that might be occurring among several species in syngameons. This will also require a combination of phylogenomic, phylogeographic and population genomic approaches on the same study group and the establishment of multiple transects across multiple geographic scales where multiple species coexist. As more whole genomes become available at the population level for tropical trees, it will be feasible to identify the genomic regions shared among the species and to understand the evolutionary mechanisms shaping the evolution of these introgressed regions. This knowledge has fundamental implications to understand the structuring of highly diverse communities in tropical regions at the ecosystem level. Future research aiming to estimate the extent of syngameons in tropical trees and the identification of the species is necessary to incorporate these networks into conservation, management and policy.

## Data Availability

This article has no additional data.

## References

[1] Mayr E. 1963 Animal species and evolution. Cambridge, UK: Belknap.

[2] Lotsy J. 1925 Species or linneon. Genetica **7**, 487–506.

[3] Gunnarsson JG. 1925 Monografi över Skandinaviens Betulae. Malmo, Sweden: Röhrs Boktryckeri.

[4] Schmitt S, Tysklind N, Derroire G, Heuertz M, Hérault B. 2021 Topography shapes the local coexistence of tree species within species complexes of Neotropical forests. Oecologia **196**, 389–398. (10.1007/s00442-021-04939-2) 33978831

[5] Schmitt S, Tysklind N, Hérault B, Heuertz M. 2021 Topography drives microgeographic adaptations of closely related species in two tropical tree species complexes. Mol. Ecol. **30**, 5080–5093. (10.1111/mec.16116) 34387001

[6] Touchette L, Godbout J, Lamothe M, Porth I, Isabel N. 2024 A cryptic syngameon within betula shrubs revealed: implications for conservation in changing subarctic environments. Evol. Appl. **17**, 1–19. (10.1111/eva.13689) PMC11022622 38633131

[7] Barnes BV, Dancik BP. 1985 Characteristics and origin of a new birch species, Betula murrayana, from southeastern Michigan. Can. J. Bot. **63**, 223–226. (10.1139/b85-025)

[8] Buck R, Flores-Rentería L. 2022 The syngameon enigma. Plants **11**, 895. (10.3390/plants11070895) 35406874 PMC9002738

[9] Cronk QC, Suarez‐Gonzalez A. 2018 The role of interspecific hybridization in adaptive potential at range margins. Mol. Ecol. **27**, 4653–4656. (10.1111/mec.14927) 30562841

[10] Suarez-Gonzalez A, Hefer CA, Lexer C, Cronk QCB, Douglas CJ. 2018 Scale and direction of adaptive introgression between black cottonwood (Populus trichocarpa) and balsam poplar (P. balsamifera). Mol. Ecol. **27**, 1667–1680. (10.1111/mec.14561) 29575353

[11] Sanderson BJ *et al*. 2023 Phylogenomics reveals patterns of ancient hybridization and differential diversification that contribute to phylogenetic conflict in willows, poplars, and close relatives. Syst. Biol. **72**, 1220–1232. (10.1093/sysbio/syad042) 37449764

[12] Wang M *et al*. 2020 Phylogenomics of the genus Populus reveals extensive interspecific gene flow and balancing selection. New Phytol. **225**, 1370–1382. (10.1111/nph.16215) 31550399

[13] Liu S *et al*. 2022 Demographic history and natural selection shape patterns of deleterious mutation load and barriers to introgression across Populus genome. Mol. Biol. Evol. **39**, msac008. (10.1093/molbev/msac008) 35022759 PMC8826634

[14] Hamilton JA, De la Torre AR, Aitken SN. 2015 Fine-scale environmental variation contributes to introgression in a three-species spruce hybrid complex. Tree Genet. Genomes **11**, 807. (10.1007/s11295-014-0817-y)

[15] Feng S, Ma H, Yin Y, Wan W, Mao K, Ru D. 2025 A complex interplay of genetic introgression and local adaptation during the evolutionary history of three closely related spruce species. Plant Divers. **47**, 620–632. (10.1016/j.pld.2025.04.007) 40734838 PMC12302640

[16] Whittemore AT, Miller RE. 2023 Dynamic properties of the pinyon pine syngameon. New Phytol. **237**, 1943–1945. (10.1111/nph.18707) 36652627

[17] Zhao W, Gao J, Hall D, Andersson BA, Bruxaux J, Tomlinson KW, Drouzas AD, Suyama Y, Wang XR. 2024 Evolutionary radiation of the Eurasian Pinus species under pervasive gene flow. New Phytol. **242**, 2353–2368. (10.1111/nph.19694) 38515228

[18] Flores-Renteria L, McElwee-Adame A, Ramadoss N, Gonzalez-Elizondo M, Sniezko R, Gonzalez-Elizondo MS. 2025 Multidirectional hybridization challenges the species barriers in North American Arbutus (Ericaceae). Glob. Ecol. Conserv. **59**, e03572. (10.1016/j.gecco.2025.e03572)

[19] Rutherford S, Rossetto M, Bragg JG, McPherson H, Benson D, Bonser SP, Wilson PG. 2018 Speciation in the presence of gene flow: population genomics of closely related and diverging Eucalyptus species. Heredity (Edinb) **121**, 126–141. (10.1038/s41437-018-0073-2) 29632325 PMC6039520

[20] McLay TGB, Fowler RM, Fahey PS, Murphy DJ, Udovicic F, Cantrill DJ, Bayly MJ. 2023 Phylogenomics reveals extreme gene tree discordance in a lineage of dominant trees: hybridization, introgression, and incomplete lineage sorting blur deep evolutionary relationships despite clear species groupings in Eucalyptus subgenus Eudesmia. Mol. Phylogenet. Evol. **187**, 107869. (10.1016/j.ympev.2023.107869) 37423562

[21] Cannon CH, Petit RJ. 2020 The oak syngameon: more than the sum of its parts. New Phytol. **226**, 978–983. (10.1111/nph.16091) 31378946

[22] Leroy T *et al*. 2020 Massive postglacial gene flow between European white oaks uncovered genes underlying species barriers. New Phytol. **226**, 1183–1197. (10.1111/nph.16039) 31264219 PMC7166129

[23] Fu R *et al*. 2022 Genome-wide analyses of introgression between two sympatric Asian oak species. Nat. Ecol. Evol. **6**, 924–935. (10.1038/s41559-022-01754-7) 35513577

[24] Eaton DAR, Hipp AL, González-Rodríguez A, Cavender-Bares J. 2015 Historical introgression among the American live oaks and the comparative nature of tests for introgression. Evolution **69**, 2587–2601. (10.1111/evo.12758) 26299374

[25] Cannon CH, Lerdau M. 2015 Variable mating behaviors and the maintenance of tropical biodiversity. Front. Genet. **6**, 183. (10.3389/fgene.2015.00183) 26042148 PMC4437050

[26] Muir EJ, Lajeunesse MJ, Kramer AM. 2024 The magnitude of Allee effects varies across Allee mechanisms, but not taxonomic groups. Oikos **2024**, 1–10. (10.1111/oik.10386)

[27] Liang YY *et al*. 2025 Pan-genome analysis reveals local adaptation to climate driven by introgression in oak species. Mol. Biol. Evol. **42** 1–21. (10.1093/molbev/msaf088) PMC12042805 40235155

[28] Suarez-Gonzalez A, Lexer C, Cronk QCB. 2018 Adaptive introgression: a plant perspective. Biol. Lett. **14**, 20170688. (10.1098/rsbl.2017.0688) 29540564 PMC5897607

[29] Cannon CH, Lerdau MT. 2019 Demography and destiny: the syngameon in hyperdiverse systems. Proc. Natl Acad. Sci. USA **116**, 8105–8105. (10.1073/pnas.1902040116) 30967502 PMC6486778

[30] Cannon CH, Lerdau M. 2022 Asking half the question in explaining tropical diversity. Trends Ecol. Evol. **37**, 392–393. (10.1016/j.tree.2022.01.006) 35177251

[31] Cooper DLM *et al*. 2024 Consistent patterns of common species across tropical tree communities. Nature **625**, 728–734. (10.1038/s41586-023-06820-z) 38200314 PMC10808064

[32] Valencia R, Condit R, Foster R, Romolerou K, Villa-Muñoz G. 2004 Yasuní forest dynamics plot, Ecuador. In Tropical forest diversity and dynamism: findings from a large-scale plot network (eds E Losos, EJ Leigh), pp. 609–620. Chicago, IL: University of Chicago Press.

[33] Ashton PS. 1969 Speciation among tropical forest trees: some deductions in the light of recent evidence. Biol. J. Linn. Soc. **1**, 155–196. (10.1111/j.1095-8312.1969.tb01818.x)

[34] Ehrendorfer F. 1970 Evolutionary patterns and strategies in seed plants. Taxon **19**, 185–195. (10.2307/1217953)

[35] Gentry AH, Gentry A. 1982 Neotropical floristic diversity: phytogeographical connections between Central and South America, pleistocene climatic fluctuations, or an accident of the andean orogeny? Ann. Mo. Bot Gard. **69**, 557. (10.2307/2399084)

[36] Abbott R *et al*. 2013 Hybridization and speciation. J. Evol. Biol. **26**, 229–246. (10.1111/j.1420-9101.2012.02599.x) 23323997

[37] Todesco M *et al*. 2016 Hybridization and extinction. Evol. Appl. **9**, 892–908. (10.1111/eva.12367) 27468307 PMC4947151

[38] Stull GW, Pham KK, Soltis PS, Soltis DE. 2023 Deep reticulation: the long legacy of hybridization in vascular plant evolution. Plant J. **114**, 743–766. (10.1111/tpj.16142) 36775995

[39] Soltis PS, Soltis DE. 2009 The role of hybridization in plant speciation. Annu. Rev. Plant Biol. **60**, 561–588. (10.1146/annurev.arplant.043008.092039) 19575590

[40] Wu CI. 2001 The genic view of the process of speciation. J. Evol. Biol. **14**, 851–865. (10.1046/j.1420-9101.2001.00335.x)

[41] Schley RJ *et al*. 2025 Rampant reticulation in a rapid radiation of tropical trees—insights from Inga (Fabaceae). Syst. Biol. syaf027. (10.1093/sysbio/syaf027) 40319371 PMC12712335

[42] Kenzo T, Kamiya K, Ngo KM, Faizu N, Lum SKY, Igarashi S, Norichika Y, Ichie T. 2019 Overlapping flowering periods among Shorea species and high growth performance of hybrid seedlings promote hybridization and introgression in a tropical rainforest of Singapore. For. Ecol. Manag. **435**, 38–44. (10.1016/j.foreco.2018.12.038)

[43] Guo X, Thomas DC, Saunders RMK. 2018 Gene tree discordance and coalescent methods support ancient intergeneric hybridisation between Dasymaschalon and Friesodielsia (Annonaceae). Mol. Phylogenet. Evol. **127**, 14–29. (10.1016/j.ympev.2018.04.009) 29678645

[44] Schley RJ, Twyford AD, Pennington RT. 2022 Hybridization: a ‘double-edged sword’ for neotropical plant diversity. Bot. J. Linn. Soc. **199**, 331–356. (10.1093/botlinnean/boab070)

[45] Gardner EM. 2023 Phylogenomic analyses of the Neotropical Artocarpeae (Moraceae) reveal a history of introgression and support the reinstatement of Acanthinophyllum. Mol. Phylogenet. Evol. **186**, 107837. (10.1016/j.ympev.2023.107837) 37270033

[46] Bacon CD, Hill A. 2024 Hybridization in palms (Arecaceae). Ecol. Evol. **14**, 1–8. (10.1002/ece3.70014) PMC11246834 39011137

[47] Linan AG, Lowry PP II, Miller AJ, Schatz GE, Sevathian J, Edwards CE. 2020 RAD‐sequencing reveals patterns of diversification and hybridization, and the accumulation of reproductive isolation in a clade of partially sympatric, tropical island trees. Mol. Ecol. **30**, 4520–4537. (10.1111/mec.15736) 33210759

[48] Larson DA, Vargas OM, Vicentini A, Dick CW. 2021 Admixture may be extensive among hyperdominant Amazon rainforest tree species. New Phytol. **232**, 2520–2534. (10.1111/nph.17675) 34389989 PMC9292926

[49] Schley RJ *et al*. 2020 Introgression across evolutionary scales suggests reticulation contributes to Amazonian tree diversity. Mol. Ecol. **29**, 4170–4185. (10.1111/mec.15616) 32881172

[50] Schmitt S. 2021 Ecological genomics of niche exploitation and individual performance in tropical forest trees. [Bordeaux, France]: Université de Bordeaux.

[51] Caron H *et al*. 2019 Chloroplast DNA variation in a hyperdiverse tropical tree community. Ecol. Evol. **9**, 4897–4905. (10.1002/ece3.5096) 31031952 PMC6476754

[52] Larson DA, Itgen MW, Denton RD, Hahn MW. 2025 Reconsidering cytonuclear discordance in the genomic age. Evolution (N Y), qpaf201. (10.1093/evolut/qpaf201) 41055410

[53] Thureborn O, Wikström N, Razafimandimbison SG, Rydin C. 2024 Plastid phylogenomics and cytonuclear discordance in Rubioideae, Rubiaceae. PLoS One **19**, e0302365. (10.1371/journal.pone.0302365) 38768140 PMC11104678

[54] Kandziora M, Sklenář P, Kolář F, Schmickl R. 2022 How to tackle phylogenetic discordance in recent and rapidly radiating groups? Developing a workflow using Loricaria (Asteraceae) as an example. Front. Plant Sci. **12**, 1–16. (10.3389/fpls.2021.765719) PMC8777076 35069621

[55] Nge FJ, Biffin E, Thiele KR, Waycott M. 2021 Reticulate evolution, ancient chloroplast haplotypes, and rapid radiation of the Australian plant genus Adenanthos (Proteaceae). Front. Ecol. Evol. **8**, 1–15. (10.3389/fevo.2020.616741)

[56] Gambhir D *et al*. 2025 Disentangling serial chloroplast captures in willows. Am. J. Bot. **112**, e70039. (10.1002/ajb2.70039) 40329507

[57] Ji Y, Yang L, Chase MW, Liu C, Yang Z, Yang J, Yang JB, Yi TS. 2019 Plastome phylogenomics, biogeography, and clade diversification of Paris (Melanthiaceae). BMC Plant Biol. **19**, 543. (10.1186/s12870-019-2147-6) 31805856 PMC6896732

[58] Low YW *et al*. 2022 Genomic insights into rapid speciation within the world’s largest tree genus Syzygium. Nat. Commun. **13**, 5031. (10.1038/s41467-022-32637-x) 36097018 PMC9468008

[59] Slik JWF *et al*. 2015 An estimate of the number of tropical tree species. Proc. Natl Acad. Sci. USA **112**, E4628–E4629. (10.1073/pnas.1423147112) 26162679 PMC4547239

[60] Condit R *et al*. 2005 Tropical tree alpha-diversity: results from a worldwide network of large plots. Biol. Skr. **55**, 565–582.

[61] Dexter KG, Lavin M, Torke BM, Twyford AD, Kursar TA, Coley PD, Drake C, Hollands R, Pennington RT. 2017 Dispersal assembly of rain forest tree communities across the amazon basin. Proc. Natl Acad. Sci. USA **114**, 2645–2650. (10.1073/pnas.1613655114) 28213498 PMC5347625

[62] Davies SJ, Palmiotto PA, Ashton PS, Lee HS, Lafrankie JV. 1998 Comparative ecology of 11 sympatric species of Macaranga in Borneo: tree distribution in relation to horizontal and vertical resource heterogeneity. J. Ecol. **86**, 662–673. (10.1046/j.1365-2745.1998.00299.x)

[63] Mori SA, Becker P, Kincaid D. 2001 Lecythidaceae of a central Amazonian lowland forest. In Lessons from amazonia: the ecology and conservation of a fragmented fores (eds ROJ Bierregaard, C Gascon, T Lovejoy, R Mesquita), pp. 54–67. New Haven, CT: Yale University Press.

[64] Arellano G, Cala V, Macía MJ. 2014 Niche breadth of oligarchic species in Amazonian and Andean rain forests. J. Veg. Sci. **25**, 1355–1366. (10.1111/jvs.12180)

[65] ter Steege H *et al*. 2013 Hyperdominance in the Amazonian tree flora. Science **342**, 1243092. (10.1126/science.1243092) 24136971

[66] Edwards DP, Davies RW, Massam MR. 2024 Ecology: a few species dominate forest tree abundance pan-tropically. Curr. Biol. **34**, R251–R254. (10.1016/j.cub.2024.02.016) 38531320

[67] Momose K *et al*. 1998 Pollination biology in a lowland dipterocarp forest in Sarawak, Malaysia. I. Characteristics of the plant-pollinator community in a lowland dipterocarp forest. Am. J. Bot. **85**, 1477–1501. 21684899

[68] Renner SS, Feil JP. 2014 Pollinators of tropical dioecious angiosperms. Am. J. Bot. **80**, 1100–1107. (10.1002/j.1537-2197.1993.tb15337.x)

[69] Bawa KS. 1990 Plant–pollinator interactions in tropical rain forests. Annu. Rev. Ecol. Syst. **21**, 399–422. (10.1146/annurev.es.21.110190.002151)

[70] Vizentin-Bugoni J, Maruyama PK, de Souza CS, Ollerton J, Rech AR, Sazima M. 2018 Plant–pollinator networks in the tropics: a review. In Ecological networks in the tropics: an integrative overview of species interactions from some of the most species-rich habitats on Earth (eds W Dáttilo, V Rico-Gray), pp. 73–91. Cham, Switzerland: Springer International Publishing. (10.1007/978-3-319-68228-0_6)

[71] Koptur S. 1984 Outcrossing and pollinator limitation of fruit set: breeding systems of Neotropical Inga trees (Fabaceae: Mimosoideae). Evolution (N Y) **38**, 1130–1143. (10.1111/j.1558-5646.1984.tb00381.x) 28555804

[72] Chen SC, Cannon CH, Kua CS, Liu JJ, Galbraith DW. 2014 Genome size variation in the Fagaceae and its implications for trees. Tree Genet. Genomes **10**, 977–988. (10.1007/s11295-014-0736-y)

[73] Heuertz M *et al*. 2020 The hyperdominant tropical tree Eschweilera coriacea (Lecythidaceae) shows higher genetic heterogeneity than sympatric Eschweilera species in French Guiana. Plant Ecol. Evol. **153**, 67–81. (10.5091/plecevo.2020.1565)

[74] Hudson CJ, Kullan ARK, Freeman JS, Faria DA, Grattapaglia D, Kilian A, Myburg AA, Potts BM, Vaillancourt RE. 2012 High synteny and colinearity among Eucalyptus genomes revealed by high-density comparative genetic mapping. Tree Genet. Genomes **8**, 339–352. (10.1007/s11295-011-0444-9)

[75] Li Y, Li X, Nie S, Zhang M, Yang Q, Xu W, Duan Y, Wang X. 2024 Reticulate evolution of the tertiary relict Osmanthus. Plant J. **117**, 145–160. (10.1111/tpj.16480) 37837261

[76] Mcguire JA *et al*. 2023 Species delimitation, phylogenomics, and biogeography of sulawesi flying lizards: a diversification history complicated by ancient hybridization, cryptic species, and arrested speciation. Syst. Biol. **72**, 885–911. (10.1093/sysbio/syad020) 37074804 PMC10405571

[77] Kauai F, Bafort Q, Mortier F, Van Montagu M, Bonte D, Van de Peer Y. 2024 Interspecific transfer of genetic information through polyploid bridges. Proc. Natl Acad. Sci. USA **121**, e2400018121. (10.1073/pnas.2400018121) 38748576 PMC11126971

[78] Bartolić P, Morgan EJ, Padilla-García N, Kolář F. 2024 Ploidy as a leaky reproductive barrier: mechanisms, rates and evolutionary significance of interploidy gene flow. Ann. Bot. **134**, 537–550. (10.1093/aob/mcae096) 38868992 PMC11523636

[79] Marburger S *et al*. 2019 Interspecific introgression mediates adaptation to whole genome duplication. Nat. Commun. **10**, 5218. (10.1038/s41467-019-13159-5) 31740675 PMC6861236

[80] Yang S, Huang J, Qu Y, Zhang D, Tan Y, Wen S, Song Y. 2024 Phylogenetic incongruence in an Asiatic species complex of the genus Caryodaphnopsis (Lauraceae). BMC Plant Biol. **24**, 616. (10.1186/s12870-024-05050-3) 38937691 PMC11212351

[81] Wanke S, Wicke S. 2023 Editorial: phylogenomic discordance in plant systematics. Front. Plant Sci. **14** 1–3. (10.3389/fpls.2023.1308126) PMC10646189 38023848

[82] de Vienne DM. 2019 Tanglegrams are misleading for visual evaluation of tree congruence. Mol. Biol. Evol. **36**, 174–176. (10.1093/molbev/msy196) 30351416

[83] Chave J, Pouchon C, Suescun U, Lavergne S, Dick C, Vargas OM, Heuertz M. 2024 Evidence for a Miocene pulse of diversification of the tropical American clade of the Brazil nut family (Lecythidaceae). Bot. Lett. **171**, 537–551. (10.1080/23818107.2024.2414981)

[84] Zwartsenberg S. 2015 Major incongruence between plastid, nuclear ribosomal and mitochondrial phylogenies in tribe Malmeeae (Annonaceae). PhD thesis. Wageningen University, Wageningen, The Netherlands.

[85] He J, Li M, Wu H, Cheng J, Xie L. 2025 Unraveling the ancient introgression history of Xanthoceras (Sapindaceae): insights from phylogenomic analysis. Int. J. Mol. Sci. **26** 1581. (10.3390/ijms26041581) 40004047 PMC11855356

[86] Karimi N, Grover CE, Gallagher JP, Wendel JF, Ané C, Baum DA. 2020 Reticulate evolution helps explain apparent homoplasy in floral biology and pollination in baobabs (Adansonia; Bombacoideae; Malvaceae). Syst. Biol. **69**, 462–478. (10.1093/sysbio/syz073) 31693158

[87] Paajanen M. 2024 Conservation and hybridization of the endangered Commidendrum–Melanodendron daisy trees of Saint Helena Island, South Atlantic ocean. PhD thesis. University of British Columbia, Vancouver, Canada.

[88] Carvalho-Madrigal S, Sanín MJ. 2024 The role of introgressive hybridization in shaping the geographically isolated gene pools of wax palm populations (genus Ceroxylon). Mol. Phylogenet. Evol. **193**, 108013. (10.1016/j.ympev.2024.108013) 38195012

[89] Reichelt N, Wen J, Pätzold C, Appelhans MS. 2021 Target enrichment improves phylogenetic resolution in the genus Zanthoxylum (Rutaceae) and indicates both incomplete lineage sorting and hybridization events. Ann. Bot. **128**, 497–510. (10.1093/aob/mcab092) 34250543 PMC8414929

[90] Chen XY, Zhou BF, Shi Y, Liu H, Liang YY, Ingvarsson PK, Wang B. 2024 Evolution of the correlated genomic variation landscape across a divergence continuum in the genus Castanopsis. Mol. Biol. Evol. **41**, 1–22. (10.1093/molbev/msae191) PMC11421576 39248185

[91] Boom AF, Migliore J, Kaymak E, Meerts P, Hardy OJ. 2022 Nuclear ribosomal phylogeny of Brachystegia: new markers for new insights about rain forests and miombo woodlands evolution. Plant Ecol. Evol. **155**, 301–314. (10.5091/plecevo.91373)

[92] Boom AF, Migliore J, Kaymak E, Meerts P, Hardy OJ. 2021 Plastid introgression and evolution of African miombo woodlands: new insights from the plastome‐based phylogeny of Brachystegia trees. J. Biogeogr. **48**, 933–946. (10.1111/jbi.14051)

[93] Boom AF, Migliore J, Ojeda D, Kaymak E, Hardy OJ. 2024 Phylogenomics of Brachystegia: insights into the origin of African miombo woodlands. Am. J. Bot. **111**, e16352. (10.1002/ajb2.16352) 38853465

[94] Fortes EA, Landis JB, Steege HT, Specht CD, Doyle JJ, Mansano V de F. 2025 Nuclear phylogenomics of Eperua (Leguminosae) highlights the role of habitat and morphological lability in dispersal and diversification across Amazonia and in the Caatinga-Cerrado ecotone. Mol. Phylogenet. Evol. **202**, 108236. (10.1016/j.ympev.2024.108236) 39549976

[95] Bruun-Lund S, Clement WL, Kjellberg F, Rønsted N. 2017 First plastid phylogenomic study reveals potential cyto-nuclear discordance in the evolutionary history of Ficus L. Mol. Phylogenet. Evol. **109**, 93–104. (10.1016/j.ympev.2016.12.031) 28042043

[96] Yan X. 2018 Echoes of ancient introgression punctuate stable genomic lineages in the evolution of figs. Proc. Natl Acad. Sci. USA **120**, 2017. (10.1073/pnas.2222035120) PMC10334730 37399402

[97] Galtier N. 2024 An approximate likelihood method reveals ancient gene flow between human, chimpanzee and gorilla. Peer Community J. **4**, e3. (10.24072/pcjournal.359)

[98] Choo LM, Loo AHB, Ang WF, Er KBH. 2022 A natural hybrid of Sindora (Fabaceae, Detarioideae) from Singapore. PhytoKeys **190**, 87–102. (10.3897/phytokeys.190.79185) 35437378 PMC8891240

[99] Schneider JV, Jungcurt T, Cardoso D, Amorim AM, Töpel M, Andermann T, Poncy O, Berberich T, Zizka G. 2021 Phylogenomics of the tropical plant family Ochnaceae using targeted enrichment of nuclear genes and 250+ taxa. Taxon **70**, 48–71. (10.1002/tax.12421)

[100] Couvreur TLP, Helmstetter AJ, Koenen EJM, Bethune K, Brandão RD, Little SA, Sauquet H, Erkens RHJ. 2019 Phylogenomics of the major tropical plant family Annonaceae using targeted enrichment of nuclear genes. Front. Plant Sci. **9**, 1941. (10.3389/fpls.2018.01941) 30687347 PMC6334231

[101] Christe C, Boluda CG, Koubínová D, Gautier L, Naciri Y. 2021 New genetic markers for Sapotaceae phylogenomics: more than 600 nuclear genes applicable from family to population levels. Mol. Phylogenet. Evol. **160**, 107123. (10.1016/j.ympev.2021.107123) 33610647

[102] Hibbins MS, Hahn MW. 2022 Phylogenomic approaches to detecting and characterizing introgression. Genetics **220**, iyab173. (10.1093/genetics/iyab173) 34788444 PMC9208645

[103] Steenwyk JL, Li Y, Zhou X, Shen XX, Rokas A. 2023 Incongruence in the phylogenomics era. Nat. Rev. Genet. **24**, 834–850. (10.1038/s41576-023-00620-x) 37369847 PMC11499941

[104] Smith SA, Moore MJ, Brown JW, Yang Y. 2015 Analysis of phylogenomic datasets reveals conflict, concordance, and gene duplications with examples from animals and plants. BMC Evol. Biol. **15**, 150. (10.1186/s12862-015-0423-0) 26239519 PMC4524127

[105] Sayyari E, Whitfield JB, Mirarab S. 2018 DiscoVista: interpretable visualizations of gene tree discordance. Mol. Phylogenet. Evol. **122**, 110–115. (10.1016/j.ympev.2018.01.019) 29421312

[106] Shang HY, Jia KH, Li NW, Zhou MJ, Yang H, Tian XL, Ma YP, Zhang RG. 2025 Phytop: a tool for visualizing and recognizing signals of incomplete lineage sorting and hybridization using species trees output from ASTRAL. Hortic. Res. **12**, uhae330. (10.1093/hr/uhae330) 40046323 PMC11879507

[107] Komarova VA, Lavrenchenko LA. 2022 Approaches to the detection of hybridization events and genetic introgression upon phylogenetic incongruence. Biol. Bull. Rev. **12**, 240–253. (10.1134/s2079086422030045)

[108] Bjorner M, Molloy EK, Dewey CN, Solis-Lemus C. 2024 Detectability of varied hybridization scenarios using genome-scale hybrid detection methods. Bull. Soc. Syst. Biol. **3**. (10.18061/bssb.v3i1.9284)

[109] Elworth RAL, Ogilvie HA, Zhu J, Nakhleh L. 2019 Advances in computational methods for phylogenetic networks in the presence of hybridization. In Bioinformatics and phylogenetics computational biology (ed. T Warnow), pp. 317–360, vol. 29. Cham, Switzerland: Springer International Publishing. (10.1007/978-3-030-10837-3_13)

[110] Rhodes JA, Baños H, Mitchell JD, Allman ES. 2021 MSCquartets 1.0: quartet methods for species trees and networks under the multispecies coalescent model in R. Bioinformatics **37**, 1766–1768. (10.1093/bioinformatics/btaa868) 33031510 PMC8289379

[111] Cai R, Ané C. 2021 Assessing the fit of the multi-species network coalescent to multi-locus data. Bioinformatics **37**, 634–641. (10.1093/bioinformatics/btaa863) 33027508

[112] Pease JB, Brown JW, Walker JF, Hinchliff CE, Smith SA. 2018 Quartet sampling distinguishes lack of support from conflicting support in the green plant tree of life. Am. J. Bot. **105**, 385–403. (10.1002/ajb2.1016) 29746719

[113] Edelman NB *et al*. 2019 Genomic architecture and introgression shape a butterfly radiation. Science **366**, 594–599. (10.1126/science.aaw2090) 31672890 PMC7197882

[114] Joly S. 2012 JML: testing hybridization from species trees. Mol. Ecol. Resour. **12**, 179–184. (10.1111/j.1755-0998.2011.03065.x) 21899723

[115] Blischak PD, Chifman J, Wolfe AD, Kubatko LS. 2018 HyDe: a Python package for genome-scale hybridization detection. Syst. Biol. **67**, 821–829. (10.1093/sysbio/syy023) 29562307 PMC6454532

[116] Pease JB, Hahn MW. 2015 Detection and polarization of introgression in a five-taxon phylogeny. Syst. Biol. **64**, 651–662. (10.1093/sysbio/syv023) 25888025

[117] Leppälä K, da Silva Coelho FA, Richter M, Albert VA, Lindqvist C. 2024 Five-leaf generalizations of the D-statistic reveal the directionality of admixture. Mol. Biol. Evol. **41**, msae198. (10.1093/molbev/msae198) 39302159 PMC11708231

[118] Patterson N, Moorjani P, Luo Y, Mallick S, Rohland N, Zhan Y, Genschoreck T, Webster T, Reich D. 2012 Ancient admixture in human history. Genetics **192**, 1065–1093. (10.1534/genetics.112.145037) 22960212 PMC3522152

[119] Hahn MW, Hibbins MS. 2019 A three-sample test for introgression. Mol. Biol. Evol. **36**, 2878–2882. (10.1093/molbev/msz178) 31373630

[120] Cai L, Xi Z, Lemmon EM, Lemmon AR, Mast A, Buddenhagen CE, Liu L, Davis CC. 2021 The perfect storm: gene tree estimation error, incomplete lineage sorting, and ancient gene flow explain the most recalcitrant ancient angiosperm clade, Malpighiales. Syst. Biol. **70**, 491–507. (10.1093/sysbio/syaa083) 33169797

[121] Elworth RAL, Allen C, Benedict T, Dulworth P, Nakhleh L. 2018 ALPHA: a toolkit for Automated Local PHylogenomic Analyses. Bioinformatics **34**, 2848–2850. (10.1093/bioinformatics/bty173) 29562324

[122] Hamlin JAP, Hibbins MS, Moyle LC. 2020 Assessing biological factors affecting postspeciation introgression. Evol. Lett. **4**, 137–154. (10.1002/evl3.159) 32313689 PMC7156103

[123] Solís-Lemus C, Bastide P, Ané C. 2017 PhyloNetworks: a package for phylogenetic networks. Mol. Biol. Evol. **34**, 3292–3298. (10.1093/molbev/msx235) 28961984

[124] Solís-Lemus C, Ané C. 2016 Inferring phylogenetic networks with maximum pseudolikelihood under incomplete lineage sorting. PLoS Genet. **12**, e1005896. (10.1371/journal.pgen.1005896) 26950302 PMC4780787

[125] Wen D, Yu Y, Zhu J, Nakhleh L. 2018 Inferring phylogenetic networks using PhyloNet. Syst. Biol. **67**, 735–740. (10.1093/sysbio/syy015) 29514307 PMC6005058

[126] Allman ES, Baños H, Rhodes JA. 2019 NANUQ: a method for inferring species networks from gene trees under the coalescent model. Algorithms Mol. Biol. **14**, 1 25. (10.1186/s13015-019-0159-2) 31827592 PMC6896299

[127] Kubatko LS. 2009 Identifying hybridization events in the presence of coalescence via model selection. Syst. Biol. **58**, 478–488. (10.1093/sysbio/syp055) 20525602

[128] Wu Y. 2013 An algorithm for constructing parsimonious hybridization networks with multiple phylogenetic trees. J. Comput. Biol. **20**, 792–804. (10.1089/cmb.2013.0072) 24093230 PMC3791036

[129] Huson DH, Scornavacca C. 2012 Dendroscope 3: an interactive tool for rooted phylogenetic trees and networks. Syst. Biol. **61**, 1061–1067. (10.1093/sysbio/sys062) 22780991

[130] Lutteropp S, Scornavacca CcDS, Kozlov AM, Morel B, Stamatakis A. 2022 NetRAX: accurate and fast maximum likelihood phylogenetic network inference. Bioinformatics **38**, 3725–3733. (10.1093/bioinformatics/btac396) 35713506 PMC9344847

[131] Flouri T, Jiao X, Rannala B, Yang Z. 2020 A Bayesian implementation of the multispecies coalescent model with introgression for phylogenomic analysis. Mol. Biol. Evol. **37**, 1211–1223. (10.1093/molbev/msz296) 31825513 PMC7086182

[132] Zhang C, Ogilvie HA, Drummond AJ, Stadler T. 2018 Bayesian inference of species networks from multilocus sequence data. Mol. Biol. Evol. **35**, 504–517. (10.1093/molbev/msx307) 29220490 PMC5850812

[133] Kong S, Swofford DL, Kubatko LS. 2025 Inference of phylogenetic networks from sequence data using composite likelihood. Syst. Biol. **74**, 53–69. (10.1093/sysbio/syae054) 39387633

[134] Müller NF, Ogilvie HA, Zhang C, Drummond AJ, Stadler T, Drummond A. 2021 Joint inference of species histories and gene flow. bioRxiv 348391. (10.1101/348391)

[135] Rabier CE, Berry V, Stoltz M, Santos JD, Wang W, Glaszmann JC, Pardi F, Scornavacca C. 2021 On the inference of complex phylogenetic networks by Markov chain Monte Carlo. PLoS Comput. Biol. **17**, e1008380. (10.1371/journal.pcbi.1008380) 34478440 PMC8445492

[136] Lipson M, Loh PR, Levin A, Reich D, Patterson N, Berger B. 2013 Efficient moment-based inference of admixture parameters and sources of gene flow. Mol. Biol. Evol. **30**, 1788–1802. (10.1093/molbev/mst099) 23709261 PMC3708505

[137] Pickrell JK, Pritchard JK. 2012 Inference of population splits and mixtures from genome-wide allele frequency data. PLoS Genet. **8**, e1002967. (10.1371/journal.pgen.1002967) 23166502 PMC3499260

[138] Huson DH. 1998 SplitsTree: analyzing and visualizing evolutionary data. Bioinformatics **14**, 68–73. (10.1093/bioinformatics/14.1.68) 9520503

[139] Huson DH, Bryant D. 2024 The SplitsTree App: interactive analysis and visualization using phylogenetic trees and networks. Nat. Methods **21**, 1773–1774. (10.1038/s41592-024-02406-3) 39223398

[140] Hibbins MS, Hahn MW. 2019 The timing and direction of introgression under the multispecies network coalescent. Genetics **211**, 1059–1073. (10.1534/genetics.118.301831) 30670542 PMC6404246

[141] Kong S, Pons JC, Kubatko L, Wicke K. 2022 Classes of explicit phylogenetic networks and their biological and mathematical significance. J. Math. Biol. **84**, 47. (10.1007/s00285-022-01746-y) 35503141

[142] Blair C, Ané C. 2020 Phylogenetic trees and networks can serve as powerful and complementary approaches for analysis of genomic data. Syst. Biol. **69**, 593–601. (10.1093/sysbio/syz056) 31432090

[143] Fitak RR. 2021 OptM: estimating the optimal number of migration edges on population trees using Treemix. Biol. Methods Protoc. **6**, bpab017. (10.1093/biomethods/bpab017) 34595352 PMC8476930

[144] Simon C, Stuckel M. 2025 Untangling a history of hybridization: a comparison of phylogenetic network methods in reconstructing reticulate evolution in New Zealand cicadas. Evol. Biol. 2025.03.04.641558. (10.1101/2025.03.04.641558)

[145] Kong S, Kubatko LS. 2021 Comparative performance of popular methods for hybrid detection using genomic data. Syst. Biol. **70**, 891–907. (10.1093/sysbio/syaa092) 33404632

[146] Hernández-Gutiérrez R, van den Berg C, Granados Mendoza C, Peñafiel Cevallos M, Freire M. E, Lemmon EM, Lemmon AR, Magallón S. 2022 Localized phylogenetic discordance among nuclear loci due to incomplete lineage sorting and introgression in the family of cotton and cacao (Malvaceae). Front. Plant Sci. **13**, 850521. (10.3389/fpls.2022.850521) 35498660 PMC9043901

[147] Hodel RGJ, Zimmer EA, Liu BB, Wen J. 2022 Synthesis of nuclear and chloroplast data combined with network analyses supports the polyploid origin of the apple tribe and the hybrid origin of the Maleae–Gillenieae clade. Front. Plant Sci. **12**, 1–15. (10.3389/fpls.2021.820997) PMC8822239 35145537

[148] Zuntini AR *et al*. 2024 Phylogenomics and the rise of the angiosperms. Nature **629**, 843–850. (10.1038/s41586-024-07324-0) 38658746 PMC11111409

[149] Zhou BF *et al*. 2022 Phylogenomic analyses highlight innovation and introgression in the continental radiations of Fagaceae across the Northern Hemisphere. Nat. Commun. **13**, 1320. (10.1038/s41467-022-28917-1) 35288565 PMC8921187

[150] Cai L, Xi Z, Amorim AM, Sugumaran M, Rest JS, Liu L, Davis CC. 2019 Widespread ancient whole‐genome duplications in Malpighiales coincide with Eocene global climatic upheaval. New Phytol. **221**, 565–576. (10.1111/nph.15357) 30030969 PMC6265113

[151] Fang Y *et al*. 2024 Pan-genome and phylogenomic analyses highlight Hevea species delineation and rubber trait evolution. Nat. Commun. **15**, 7232. (10.1038/s41467-024-51031-3) 39174505 PMC11341782

[152] Zhu XL, Luo WJ, Chai SF, Tang JM, Wei X, Wee AKS, Kang M. 2025 Potential risk of dipterocarps in the marginal Asian rainforests: low population size and high genomic erosion. BMC Biol. **23**, 161. (10.1186/s12915-025-02275-y) 40484938 PMC12147322

[153] Tricou T, Tannier E, de Vienne DM. 2022 Ghost lineages highly influence the interpretation of introgression tests. Syst. Biol. **71**, 1147–1158. (10.1093/sysbio/syac011) 35169846 PMC9366450

[154] Shang H *et al*. 2023 Drivers of genomic landscapes of differentiation across a Populus divergence gradient. Mol. Ecol. **32**, 4348–4361. (10.1111/mec.17034) 37271855

[155] Buck R, Ortega‐Del Vecchyo D, Gehring C, Michelson R, Flores‐Rentería D, Klein B, Whipple AV, Flores‐Rentería L. 2023 Sequential hybridization may have facilitated ecological transitions in the Southwestern pinyon pine syngameon. New Phytol. **237**, 2435–2449. (10.1111/nph.18543) 36251538

[156] Ma XG, Ren YB, Sun H. 2024 Introgression and incomplete lineage sorting blurred phylogenetic relationships across the genomes of sclerophyllous oaks from southwest China. Cladistics **40**, 357–373. (10.1111/cla.12570) 38197450

[157] Forest P *et al*. 2021 Taking the pulse of Earth’s tropical forests using networks of highly distributed plots. Biol. Conserv. **260**, 108849. (10.1016/j.biocon.2020.108849)

[158] Tysklind N *et al*. 2020 Microgeographic local adaptation and ecotype distributions: the role of selective processes on early life‐history traits in sympatric, ecologically divergent Symphonia populations. Ecol. Evol. **10**, 10735–10753. (10.1002/ece3.6731) 33072293 PMC7548183

[159] Gilles D *et al*. 2016 RAINBIO: a mega-database of tropical African vascular plants distributions. PhytoKeys **74**, 1–18. (10.3897/phytokeys.74.9723) PMC5234546 28127234

[160] Cannon CH, Scher CL. 2017 Exploring the potential of gametic reconstruction of parental genotypes by F1 hybrids as a bridge for rapid introgression. Genome **60**, 713–719. (10.1139/gen-2016-0181) 28732173

[161] Rosenzweig BK, Pease JB, Besansky NJ, Hahn MW. 2016 Powerful methods for detecting introgressed regions from population genomic data. Mol. Ecol. **25**, 2387–2397. (10.1111/mec.13610) 26945783 PMC4899106

[162] He Z, Li X, Yang M, Wang X, Zhong C, Duke NC, Wu CI, Shi S. 2019 Speciation with gene flow via cycles of isolation and migration: insights from multiple mangrove taxa. Natl. Sci. Rev. **6**, 275–288. (10.1093/nsr/nwy078) 31258952 PMC6599600

[163] Laetsch DR, Bisschop G, Martin SH, Aeschbacher S, Setter D, Lohse K. 2023 Demographically explicit scans for barriers to gene flow using gIMble. PLoS Genet. **19**, e1010999. (10.1371/journal.pgen.1010999) 37816069 PMC10610087

[164] Zhang H, Zhang X, Wu G, Dong C, Liu J, Li M. 2023 Genomic divergence and introgression among three Populus species. Mol. Phylogenet. Evol. **180**, 107686. (10.1016/j.ympev.2022.107686) 36586545

[165] Dagilis AJ, Matute DR. 2023 The fitness of an introgressing haplotype changes over the course of divergence and depends on its size and genomic location. PLoS Biol. **21**, e3002185. (10.1371/journal.pbio.3002185) 37459351 PMC10374083

[166] Schrider DR, Ayroles J, Matute DR, Kern AD. 2018 Supervised machine learning reveals introgressed loci in the genomes of Drosophila simulans and D. sechellia. PLoS Genet. **14**, e1007341. (10.1371/journal.pgen.1007341) 29684059 PMC5933812

[167] Gower G, Picazo PI, Fumagalli M, Racimo F. 2021 Detecting adaptive introgression in human evolution using convolutional neural networks. eLife **10**, 1–45. 64669. (10.7554/elife.64669) PMC8192126 34032215

[168] Zhang Y, Zhu Q, Shao Y, Jiang Y, Ouyang Y, Zhang L, Zhang W. 2023 Inferring historical introgression with deep learning. Syst. Biol. **72**, 1013–1038. (10.1093/sysbio/syad033) 37257491

[169] Ray DD, Flagel L, Schrider DR. 2024 IntroUNET: identifying introgressed alleles via semantic segmentation. PLoS Genet. **20**, e1010657. (10.1371/journal.pgen.1010657) 38377104 PMC10906877

[170] Setter D, Mousset S, Cheng X, Nielsen R, DeGiorgio M, Hermisson J. 2020 VolcanoFinder: genomic scans for adaptive introgression. PLoS Genet. **16**, e1008867. (10.1371/journal.pgen.1008867) 32555579 PMC7326285

[171] Jackiw RN, Mandil G, Hager HA. 2015 A framework to guide the conservation of species hybrids based on ethical and ecological considerations. Conserv. Biol. **29**, 1040–1051. (10.1111/cobi.12526) 25976359

[172] Narcisse E, Njila N, Libalah MB, Medou N, Ngoukwa G, Amidou PM, Issa M. 2025 Biodiversity and conservation status of Gilbertiodendron dewevrei monodominant forests and adjacent mixed forests in the Congo basin. Env. Anal Ecol Stud **13**, 1591–1609. (10.31031/EAES.2025.13.000803)

[173] Heimpel E, Ahrends A, Dexter KG, Hall JS, Mamboueni J, Medjibe VP, Morgan D, Sanz C, Harris DJ. 2024 Floristic and structural distinctness of monodominant Gilbertiodendron dewevrei forest in the western Congo Basin. Plecevo **157**, 55–74. (10.5091/plecevo.111539)

[174] Cannon CH. 2017 Assisted diversification for an era of habitat extinction. Gene Conservation of Tree Species—Banking on the Future. Proceedings of a workshop. Gen. Tech. Rep. PNW-GTR-963, p. 71. Portland, OR: U.S. Department of Agriculture, Forest Service, Pacific Northwest Research Station.

[175] Reutimann O, Baltensweiler A, Walthert L, Olofsson JK, Zimmerman F, Olofsson J. 2025 Fine-scale variation in soil properties promotes local taxonomic diversity of hybridizing oak species (Quercus spp.). Evol. Appl. **18**, e70076. (10.1111/eva.70076) 39925616 PMC11802334

[176] Broyles SB. 2002 Hybrid bridges to gene flow: a case study in milkweeds (Asclepias). Evolution **56**, 1943–1953. (10.1111/j.0014-3820.2002.tb00120.x) 12449481

